# Changes in Antioxidant and Photosynthetic Capacity in Rice Under Different Substrates

**DOI:** 10.3390/biology14010034

**Published:** 2025-01-04

**Authors:** Hang Zhou, Liming Zhao, Yiwen Song, Xiaole Du, Jingxin Huo, Wanqi Mei, Xi Wang, Naijie Feng, Dianfeng Zheng, Zhaohui Wu

**Affiliations:** 1 School of Tropical Agriculture and Forestry, Hainan University, Haikou 570100, China; 2 College of Coastal Agricultural Sciences, Guangdong Ocean University, Zhanjiang 524000, China; 3 National Saline-Alkali Tolerant Rice Technology Innovation Center, Sanya 572000, China; 4 National Key Laboratory of Hybrid Rice, Hunan Hybrid Rice Research Center, Changsha 410125, China

**Keywords:** rice, soil, antioxidant, photosynthesis

## Abstract

Against the backdrop of a changing global climate, the soil environment may undergo significant changes, directly affecting agricultural productivity and exacerbating global food security issues. Three different substrates were set up in this study, namely, S (high sand and low nutrient content), T (medium sand and medium nutrient content), and TT (low sand and high nutrient content). The results showed that the root/shoot ratio increased as the sand content increased (nutrient content decreased). Rice in different substrates had various degrees of dependence on antioxidant enzymes and antioxidants. For example, seedlings in TT treatment may depend more on ascorbic acid (AsA) compared to T. In addition, compared with S and T, the photosynthetic activity of rice in the optimized substrate (TT) was the highest; the net photosynthetic rate (Pn) of TT seedlings was significantly higher than that of T. This study also detected that the change in substrates affected the gas exchange parameters of rice leaves. The transpiration rate (Tr) and stomatal conductance (Gs) of the TT treatment were higher than those of the T treatment. The results of this study may provide a scientific basis for formulating agricultural management strategies.

## Simple Summary

Climate change is a complex global challenge facing humanity. Rising global temperatures lead to increased evaporation and soil moisture deficit, causing degradation of agricultural land and desertification. This study explored the changes in rice dry weight, antioxidant enzyme activity, antioxidant content, and photosynthetic characteristics under different substrate conditions (S: high sand and low nutrient content; T: medium sand and medium nutrient content; TT: low sand and high nutrient content), and preliminarily revealed the physiological responses of rice seedlings to changing substrate conditions. The results of this study provide theoretical support for optimizing agricultural production strategies and ensuring global food security against a background of global climate change.

## 1. Introduction

Rice is an important food crop all over the world, and the populations of many countries depend on rice as the primary food source, especially in Asia. In addition to being a food crop, rice is an essential agricultural cash crop in many countries, which use it as an important export commodity.

Climate change is a complex global challenge facing humanity, affecting multiple fields, including ecology, environment, socio-politics, and socio-economics [1,2,3,4]. According to reports, the Earth’s temperature in 2022 was about 2 degrees Fahrenheit (about 1.11 degrees Celsius) higher than the average temperature at the end of the 19th century, and the Earth will continue its long-term warming trend (https://svs.gsfc.nasa.gov/5060/) (accessed on 15 October 2024).

Biological processes in soil are controlled to a large extent by soil temperature and moisture [5,6]. A strong negative correlation exists between soil organic carbon (SOC) and temperature [7,8,9,10,11]. Increased temperatures stimulate microbial activity, thereby improving soil organic matter (SOM) decomposition [11]. SOC plays a role in carbon cycling in terrestrial ecosystems [12,13] since SOC makes up 48–58% of the weight of SOM [13,14]. SOC is an acknowledged indicator of land degradation [15], and it directly determines soil quality and food production [16]. A global meta-analysis by Oldfield et al. (2019) indicated potential yield increases of 10% ± 11% for maize and 23% ± 37% for wheat with increase in SOM content [17,18]. Ghaley et al. (2018) found that SOC level significantly affected the grain yield of wheat and aboveground biomass at 0 to 100 kg N ha^−1^ [18,19]. In addition, rising global temperatures will also lead to increased evaporation and soil moisture deficit, causing degradation of agricultural land and desertification. Vegetation growth is sensitive to temperature changes; high temperatures during the growing season can affect vegetation growth, exacerbating desertification [20]. Soil is the foundation of agricultural production and the most precious natural resource on earth. It provides plants with essential nutrients and water and supports agricultural development. Against the backdrop of a changing global climate, the soil environment may undergo significant changes, directly affecting agricultural productivity and exacerbating global food security issues.

Climate change may cause changes in various soil characteristics, such as soil texture and nutrient content, affecting the growth of plants growing in the soil. This study set up three substrates by adjusting the ratio of sand to soil, revealing the effects of substrate changes on rice growth and adaptability, which may provide a scientific basis for formulating agricultural management strategies.

## 2. Materials and Methods

### 2.1. Experimental Design

The experiment was conducted in Guangdong Ocean University in 2022–2023. The tested variety is Huanghuazhan, with a growth period of 139.8 days. In this study, 3 different substrates were set up, namely, S (inland beach sand), T (inland beach sand:brick red soil:peat soil = 10:1:1), and TT (inland beach sand:brick red soil:peat soil = 3:1:1). The physical and chemical properties of the substrates were shown in Table 1. Sterilized seeds were soaked in distilled water for 24 h, followed by germination for 24 h. The germinated seeds were sown in flower pots of 20.4 cm × 16.9 cm × 14.5 cm (upper diameter, height, lower diameter), and 57 plants were evenly sown in each pot (each pot contained the same volume of substrate).

### 2.2. Determination of Dry Weight

The dry weights of the shoots and roots were measured with an analytical balance on the 17th and 19th days after sowing. The root/shoot ratio was calculated according to the following formula [21]:

Root/shoot ratio = Root dry weight/shoot dry weight

### 2.3. Determination of Antioxidant Enzyme Activity

In this study, superoxide dismutase (SOD), peroxidase (POD), catalase (CAT), and ascorbate peroxidase (APX) activities were determined according to the method of Zhang et al. (2023) [22].

### 2.4. Determination of AsA and Glutathione (GSH) Content

The AsA content was measured according to the methods of Chen et al. (2022) [23] and Huang et al. (2023) [24]. The GSH content was measured according to the methods of Yan et al. (2021) [25] and Huang et al. (2023) [24].

### 2.5. Determination of Photosynthesis-Related Parameters

A portable Li6800 photosynthetic instrument was used to determine the Pn, Gs, Tr, and intercellular carbon dioxide concentration (Ci) of the latest fully expanded leaves (9 a.m. to 11 a.m.).

### 2.6. Chlorophyll Fluorescence Detection

After 30 min of dark treatment, the latest fully expanded rice leaves were selected for chlorophyll fluorescence detection using a portable modulated chlorophyll fluorescence instrument (PAM-2500, Heinz Walz, Germany).

### 2.7. Determination of Physical and Chemical Parameters of Substrates

The physical and chemical parameters of substrates were determined according to the method of Bao (2000) [26].

#### 2.7.1. Determination of SOM and SOC

A quantity of 0.1 g of the substrate sample passed through a 60-mesh sieve (<0.25 mm) was placed in a hard test tube; 2 mL of 0.8 mol·L^−1^ potassium dichromate and 2 mL of concentrated sulfuric acid were accurately added to the test tube, and then a small funnel (or small glass bubble) was covered on the test tube mouth. The test tubes were then placed in an oven at 160 °C for 30 min. The funnel was rinsed with distilled water, the rinse was dripped into the test tube, and additional water was added to about 10 mL. Two drops of the o-phenanthroline indicator were added and titrated with the prepared ferrous sulfate solution using a semi-microburette. The solution changed from yellow to green, light green, and suddenly turned into coffee red as the end point. The blank test process was the same as above. The SOM content was calculated as follows:

SOM content (g·kg^−1^) = [0.8000 × 2/V0 × (V0 − V1) × 0.003 × 1.724 × 1.1]/sample weight × 1000

V0 is the volume (mL) of ferrous sulfate used when titrating the blank solution; V is the volume (mL) of ferrous sulfate used when titrating the sample; 0.003 is 1/4 millimolar mass of carbon; 1.1 is oxidation calibration constant; 1.724 is conversion coefficient for SOC and SOM.

#### 2.7.2. Determination of Alkaline Nitrogen

Two grams of air-dried substrate sample was passed through a 1 mm sieve, and a teaspoon of ferrous sulfate powder was evenly spread in the outer chamber of the diffusion dish. The diffuser dish was gently rotated horizontally to flatten the soil sample. A 2 mL quantity of 2% boric acid solution containing indicator was added to the inner chamber of the diffusion vessel. The outer chamber rim of the dish was then coated with alkaline glycerol. The ground glass was covered. A 10 mL quantity of 1.8 mol·L^−1^ NaOH solution was quickly added to the outer chamber of the diffuser dish, and the frosted glass was immediately rotated and covered tightly. The diffuser dish was gently rotated horizontally to mix the solution with the soil fully and then placed in a 40 °C incubator. It was taken out after 24 h, and the amount of ammonia absorbed by the boric acid solution in the inner chamber of the diffusion dish was titrated with a microburette with 0.005 mol·L^−1^ H_2_SO_4_ standard solution, and the end point was purple. Another diffusion dish was used as a blank test without adding soil, and the other steps were the same.

#### 2.7.3. Determination of Rapidly Available Potassium

A 5 g quantity of the air-dried substrate sample passed through a 2 mm sieve was placed in a 100 mL plastic bottle, and 50.0 mL of 1 mol·L^−1^ ammonium acetate solution was added (the ratio of substrates to liquid was 1:10). Then, the plastic bottle was shaken for 15 min, and the mixture was filtered. The zero point of the instrument was adjusted with the 0 um·mL^−1^ solution of the standard solution, and the filtrate was directly measured on the flame photometer.

#### 2.7.4. Determination of pH

The air-dried substrate sample was ground and passed through a 2 mm sieve, and 10.0 g of the sample was weighed in a 50 mL beaker. A 25 mL quantity of carbon dioxide-free distilled water, 1 mol·L^−1^ potassium chloride solution (acid soil), or 0.01 mol·L^−1^ calcium chloride solution (neutral and alkaline soil) was added. The mixture was vigorously stirred with a glass rod for 1–2 min and left to stand for 30 min. The pH value was measured using a pH meter according to the instrument instructions.

### 2.8. Statistical Analysis

This study used SPSS 27 to perform a one-way analysis of variance and independent sample T-test and Origin 2021 to draw figures.

## 3. Result

### 3.1. Dry Weight and Root/Shoot Ratio

**Figure 1:**
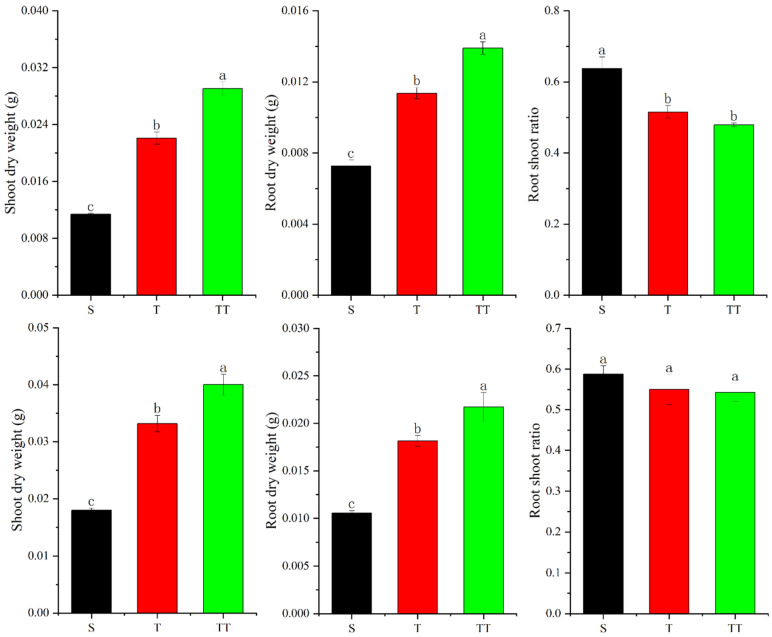
Changes in rice dry weight and root/shoot ratio in different substrates. The first row is the first measurement result; the second row is the second measurement result. The different letters above the bars indicate statistically significant differences.

As shown in Figure 1 below, the dry weight of rice shoots and roots changed regularly; that is, S < T < TT, indicating that the changes in substrates caused differences in rice growth. At the same time, this study observed that the root/shoot ratio showed a regular change of S > T > TT, indicating that the S treatment was more inclined to develop roots than other treatments.

In addition, this study conducted correlation analysis on the shoots, roots, and root/shoot ratio with physical and chemical parameters of substrates, as shown in Table 2. This study found that the dry weight of rice (shoot or root) was positively correlated with rapidly available potassium, alkaline nitrogen, organic matter, organic carbon, total nitrogen, and C/N ratio and negatively correlated with pH; among them, shoot or root dry weight was significantly positively correlated with C/N ratio (*p* ≥ 0.998 *). In contrast, the root/shoot ratio was positively correlated with pH but negatively correlated with other substrate parameters.

### 3.2. Antioxidant System

**Figure 2:**
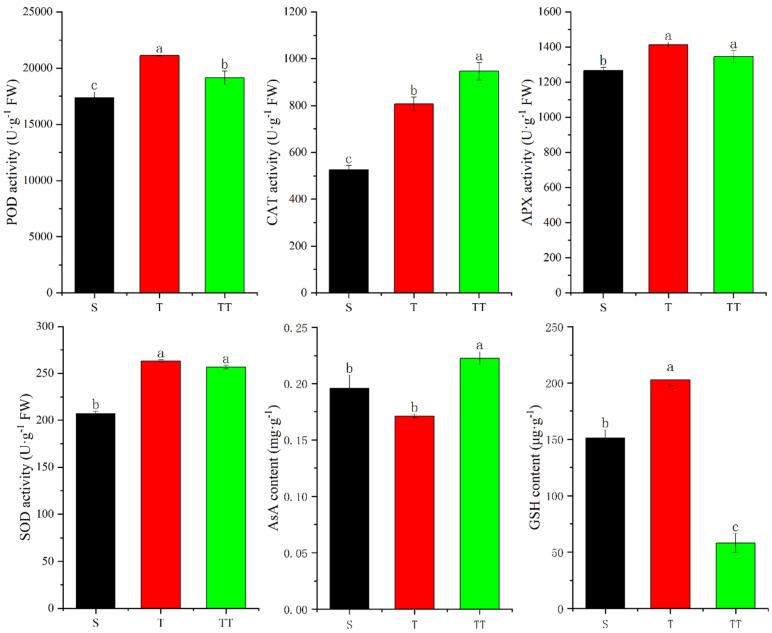
Changes in antioxidant enzyme activity and antioxidant content in different substrates. The different letters above the bars indicate statistically significant differences.

As shown in Figure 2 below, the POD activity of T was significantly higher than that of S and TT; TT was significantly higher than S. The changing pattern of CAT activity was different from that of POD. The CAT activity of TT was significantly higher than that of T and S; at the same time, the activity of T was significantly higher than that of S. For another antioxidant enzyme, APX, the activities of T and TT were significantly higher than that of S; there was no significant difference between T and TT. The changing pattern of SOD activity was same as that of APX; that is, the activities of T and TT were significantly higher than that of S, but there was no significant difference between T and TT. In addition, as essential components in the plant antioxidant system, the contents of AsA and GSH changed. For AsA, the content of TT was significantly higher than that of T and S. The GSH content of T was significantly higher than that of S and TT; S was significantly higher than TT.

Further correlation analysis showed that GSH content was positively correlated with pH but negatively correlated with rapidly available potassium, alkaline nitrogen, organic matter, organic carbon, total nitrogen, and C/N ratio. POD, CAT, APX, SOD activity, and AsA content were negatively correlated with pH and positively correlated with other substrate parameters; among them, CAT was extremely significantly positively correlated with the C/N ratio (*p* = 1 **) (Table 3).

### 3.3. Chlorophyll Fluorescence Detection

**Figure 3:**
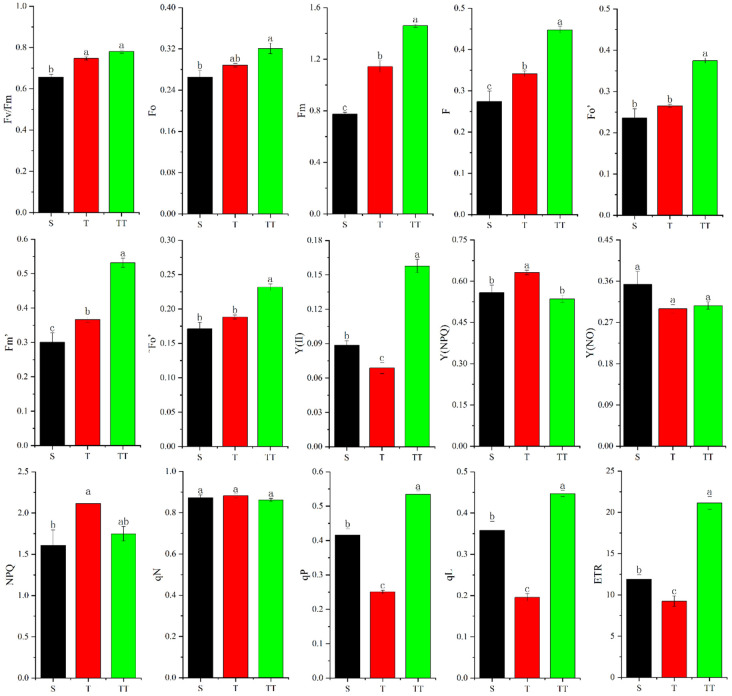
Changes in chlorophyll fluorescence parameters in different substrates. Fv/Fm: maximum photosynthetic quantum yield of PS II, reflecting the photosynthetic potential of the sample. Fo: minimum fluorescence yield. Fm: maximum fluorescence yield. F: actual fluorescence intensity at any time. Fo’: minimum fluorescence yield under light. Fm’: maximum fluorescence yield under light. ^~^Fo’: minimum fluorescence under light. Y(II): actual photosynthetic quantum yield of PS II. Y(NPQ): the quantum yield of regulatory energy dissipation. Y(NO): the quantum yield of nonregulatory energy dissipation. NPQ: non-photochemical quenching coefficient. qN: non-photochemical quenching coefficient. qP: photochemical quenching coefficient. qL: photochemical quenching coefficient. ETR: electron transfer rate through PS II. The different letters above the bars indicate statistically significant differences.

In this study, Fv/Fm, Fo, Fm, F, Fo’, Fm’, and ^~^Fo’ in rice seedling leaves showed a decreasing trend, i.e., S < T < TT; TT treatment showed the maximum values of these parameters. For Y(II), qP, qL, and ETR, the three treatments showed T < S < TT; TT was significantly higher than T and S, and S was significantly higher than T. In addition, T had the highest Y(NPQ), significantly higher than S and TT. The NPQ of T was also higher than that of TT and S, and the difference between S and T was significant (Figure 3).

The results of correlation analysis showed that rapidly available potassium was negatively correlated with Y(NPQ), Y(NO), and qN, respectively, and positively correlated with Fv/Fm, Fo, Fm, F, Fo’, Fm’, ^~^Fo’, Y(Ⅱ), NPQ, qP, qL, and ETR, respectively; among them, Fm’ and ^~^Fo’ were extremely significantly positively correlated with rapidly available potassium (*p* = 1 **). Similarly, Y(NPQ), Y(NO), and qN were negatively correlated with alkaline hydrolyzed nitrogen, and the other indicators were positively correlated with alkaline hydrolyzed nitrogen; among them, Fo (*p* = 0.998 *) and Fm (*p* = 0.997 *) were significantly positively correlated with alkali hydrolyzed nitrogen. The difference was that, except for Y(NPQ), Y(NO), and qN, other indicators were negatively correlated with pH; among them, Fm was significantly negatively correlated with pH (*p* = −1 *). In addition, the correlations of organic matter, organic carbon, total nitrogen, and C/N ratio with these chlorophyll fluorescence indicators showed similarities; except for Y(NPQ), Y(NO), and qN, other indicators were positively correlated with organic matter, organic carbon, total nitrogen, and C/N ratio; Fo was significantly positively correlated with organic matter, organic carbon, and total nitrogen (*p* = 0.999 *); similarly, F was significantly positively correlated with organic matter (*p* = 0.998 *), organic carbon (*p* = 0.998 *), and total nitrogen (*p* = 0.997 *); there was a significant positive correlation between Fv/Fm and C/N ratio (*p* = 0.997 *) (Table 4).

### 3.4. Photosynthesis Related Indicators

**Figure 4:**
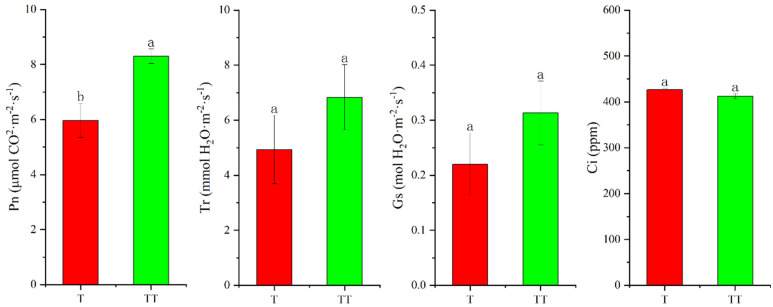
Photosynthesis parameters. Pn: net photosynthetic rate; Tr: transpiration rate; Gs: stomatal conductance; Ci: intercellular carbon dioxide concentration. The different letters above the bars indicate statistically significant differences.

Furthermore, this study detected Pn, Tr, Gs, and Ci of rice seedling leaves. As shown in Figure 4 below, Pn, Tr, and Gs of TT were all higher than those of T. The change of substrate had little effect on Ci.

## 4. Discussion

Under normal conditions, reactive oxygen species (ROS) produced by plants mainly come from photosynthesis, photorespiration, and respiration [27]. Currently, the level of ROS produced in the cell is low. However, ROS levels increase when subjected to abiotic stress, activating stress pathways within plant cells [28]. At low concentrations, ROS can act as signaling molecules, inducing the expression of defense genes and regulating the adaptive response in plants [29]. Excessive accumulation of ROS is harmful to plants [30]. In order to cope with the oxidative stress caused by ROS, higher plants have developed a complex clearance system, including enzymatic and non-enzymatic systems [31]. Antioxidant enzymes can convert excess ROS and free radicals in plants into less toxic or harmless substances, balancing the plant’s reactive oxygen content. SOD is a key enzyme in the plant antioxidant system. It can remove superoxide anions and generate H_2_O_2_ and O_2_; subsequently, CAT and APX catalyze H_2_O_2_ to generate water and divalent oxygen [32,33]. To explore the impact of substrate changes on the antioxidant system of rice, this study measured the activities of SOD, POD, CAT, and APX in rice leaves. This study found that there was no significant difference in SOD and APX activities between T and TT; compared with these two treatments, the SOD and APX activities of S were significantly reduced. Due to their essential role in maintaining redox balance within plant cells, increased activity helps protect cells from oxidative damage in T and TT. CAT is another essential antioxidant enzyme. In this study, the pattern of CAT activity showed TT > T > S, and there were significant differences between different treatments. Since the TT treatment had the lowest sand content and the highest nutrient content, this may have provided conditions for the biosynthesis of CAT. Compared with the CAT activity in TT, the activity in T was second, and S was the worst. A series of limiting factors in the T and S treatments led to a decrease in CAT activity, which in turn affected the decomposition process of H_2_O_2_. According to a previous report [34], POD, CAT, and SOD were all positively correlated with N, which was consistent with the results of this study. Moreover, according to the results of correlation analysis, this study found that the activities of the four antioxidant enzymes were positively correlated with the C/N ratio. Compared with other soil parameters, the correlation coefficient between antioxidant enzyme activity and C/N ratio was the largest, which suggested a close relationship between rice antioxidant enzyme activity and soil C/N ratio. This discovery helps guide how to deal with the relationship between scientific fertilization and crop stress resistance. In addition, this study also detected that the POD activity of S was significantly lower than that of T and TT. POD is another enzyme that catalyzes the decomposition of H_2_O_2_ into oxygen and water. Different from the change pattern of CAT activity, the POD activity of T was significantly higher than that of TT.

In addition to antioxidant enzymes, some non-enzymatic antioxidants in plants also play an essential role under abiotic stress. GSH and AsA are representatives of non-enzymatic antioxidants in plants. AsA and GSH with high redox potentials can interact with many components and pathways towards the maintenance of a generally reduced state [35]. In this study, the AsA content of seedlings under TT treatment was the highest, and that of T was the lowest. However, the trend of GSH content was just the opposite, with the lowest GSH content in seedlings under TT treatment and the highest in T. Such results indicated that rice had different requirements for these two antioxidants under different substrates. Since TT had the lowest sand content and the highest nutrient content, the seedlings at this condition may be more dependent on AsA than GSH, which proved that the antioxidant strategies of this rice variety under different substrates were different.

Fv/Fm is a measure of plant photosynthetic activity, and reduced values possibly indicate stress, photoinhibition, and photosynthesis downregulation [36]. Fv/Fm gives a robust indicator of the maximum quantum yield of PSII chemistry [37,38,39]. In this study, the Fv/Fm of TT was the highest, which means that rice had the highest photosynthetic activity under TT conditions. Compared with TT and T, the Fv/Fm of rice under S treatment was the lowest, indicating that the efficiency of rice PSII in converting absorbed light energy into chemical energy was the lowest under this state. Furthermore, Fv/Fm was negatively correlated with pH (*p* = −0.973) and significantly positively correlated with C/N ratio (*p* = 0.997 *). Since too high or too low pH is not conducive to plant growth, we speculated that appropriately lowering the pH may be more conducive to improving the photosynthetic activity of rice. Meanwhile, the strong positive correlation between Fv/Fm and C/N ratio may imply that managing soil C/N ratio is a possible way to optimize the photosynthetic efficiency of rice. Therefore, regulating the soil C/N ratio through precise fertilization to optimize photosynthetic efficiency contributes to high rice yield and sustainable agricultural development.

qP is used to indicate photoinhibition and determine the fluorescence’s level of photoprotective quenching [40]. The results of this study showed that the qP of TT was the largest compared with other treatments, which means that in the light adaptation state, the ability of PSII of rice under TT conditions to carry out photochemical reactions was the strongest. Surprisingly, this ability of rice under T treatment was weaker than that of rice under S conditions, because the qP of T was significantly lower than that of S. We speculated that the extreme conditions of S treatment may trigger the response mechanism of this rice variety, allowing for it to maintain a higher qP compared to T.

NPQ is the non-photochemical quenching parameter. Non-photochemical quenching is a mechanism used by plants to protect themselves when the light is too strong, reflecting the ability of plants to dissipate excess light energy as heat; that is, photoprotection ability. The non-photochemical quenching process acts as a “safety valve” to dissipate excess energy as heat and prevent oxidative damage [41,42,43]. In this study, the NPQ of rice was the largest under the T treatment, indicating that under this condition, the ability of rice to dissipate excess light energy as heat was the strongest. The TT treatment had the highest nutrient content and the lowest sand content in the substrate, but the NPQ of TT did not show an advantage compared with T.

Y(NO) refers to the quantum yield of non-regulated energy dissipation at PSII and is an essential indicator of photodamage. The results of this study found that rice Y(NO) under S treatment was slightly higher than that under T and TT, which indicated that compared with other treatments, the photochemical energy conversion and protective regulatory mechanisms of S seedlings may not be sufficient to consume the light energy absorbed by the plants completely. In other words, since the Y(NO) of S seedlings was the highest among the three treatments, S seedlings were most likely to be damaged.

Based on the detection results of chlorophyll fluorescence, this study also measured various photosynthesis parameters. Pn of TT treatment was significantly higher than that of T, which was consistent with the measurement results of Fv/Fm. In addition, the results showed that the Tr and Gs of TT were higher than those of T, indicating that substrate optimization (i.e., reduced sand content and increased nutrient content) can affect the gas exchange of rice leaves, which is conducive to absorbing more carbon dioxide and increasing the photosynthesis rate.

## 5. Conclusions

This study initially investigated the physiological responses of rice seedlings to changing substrates. The antioxidant strategies of rice seedlings were different under different substrates. In addition, as the sand content decreased (nutrient content increased), photosynthetic activity also increased, but it did not cause a sustained increase in the non-photochemical quenching parameter NPQ. The results of this study provide theoretical support for optimizing agricultural production strategies and ensuring global food security against a background of global climate change.

## Figures and Tables

**Table 1:** Physical and chemical properties of substrates.

	Rapidly Available Potassium (mg·kg^−1^)	Alkaline Nitrogen (mg·kg^−1^)	pH	Organic Matter (g·kg^−1^)	Organic Carbon (g·kg^−1^)	Total Nitrogen (g·kg^−1^)	C/N Ratio
S	4.63	2.45	8.54	2.11	1.22	0.11	10.78
T	11.92	20.30	7.72	25.80	14.96	1.36	11.03
TT	30.14	40.25	6.97	55.36	32.11	2.88	11.16

**Table 2:** Correlation analysis of the physical and chemical parameters of substrates with rice dry weight and root/shoot ratio.

	RAP	AN	pH	OM	OC	TN	CN
Shoot1	0.934	0.988	−0.995	0.983	0.983	0.984	0.998 *
Root1	0.929	0.986	−0.994	0.98	0.98	0.982	0.999 *
RS1	−0.855	−0.944	0.962	−0.933	−0.933	−0.936	−0.992
Shoot2	0.897	0.97	−0.982	0.961	0.961	0.963	0.999 *
Root2	0.903	0.973	−0.984	0.965	0.965	0.967	1.000 *
RS2	−0.815	−0.918	0.939	−0.905	−0.905	−0.908	−0.981

RAP: rapidly available potassium; AN: alkaline nitrogen; OM: organic matter; OC: organic carbon; TN: total nitrogen; CN: C/N ratio; Shoot1: shoot dry weight at first sampling; Root1: root dry weight at first sampling; RS1: root/shoot ratio at first sampling; Shoot2: shoot dry weight at second sampling; Root2: root dry weight at second sampling; RS2: root/shoot ratio at second sampling. * *p* < 0.05.

**Table 3:** Correlation analysis of the physical and chemical parameters of substrates with antioxidant enzyme activity and antioxidant content.

	RAP	AN	pH	OM	OC	TN	CN
POD	0.245	0.442	−0.493	0.414	0.414	0.421	0.621
CAT	0.908	0.975	−0.987	0.968	0.968	0.97	1.000 **
APX	0.327	0.518	−0.566	0.49	0.49	0.497	0.686
SOD	0.64	0.787	−0.821	0.767	0.767	0.771	0.899
AsA	0.713	0.55	−0.501	0.576	0.577	0.57	0.362
GSH	−0.8	−0.658	0.613	−0.681	−0.681	−0.676	−0.484

RAP: rapidly available potassium; AN: alkaline nitrogen; OM: organic matter; OC: organic carbon; TN: total nitrogen; CN: C/N ratio. ** *p* < 0.01.

**Table 4:** Correlation analysis of the physical and chemical parameters of substrates with chlorophyll fluorescence parameters.

	RAP	AN	pH	OM	OC	TN	CN
Fv/Fm	0.877	0.958	−0.973	0.949	0.949	0.951	0.997 *
Fo	0.989	0.998 *	−0.992	0.999 *	0.999 *	0.999 *	0.962
Fm	0.959	0.997 *	−1.000 *	0.994	0.994	0.995	0.991
F	0.994	0.995	−0.988	0.998 *	0.998 *	0.997 *	0.953
Fo’	0.997	0.958	−0.94	0.967	0.967	0.965	0.877
Fm’	1.000 **	0.978	−0.964	0.984	0.984	0.983	0.912
^~^Fo’	1.000 **	0.977	−0.963	0.983	0.983	0.982	0.91
Y(II)	0.879	0.76	−0.721	0.78	0.78	0.776	0.606
Y(NPQ)	−0.45	−0.253	0.197	−0.284	−0.284	−0.277	−0.044
Y(NO)	−0.632	−0.78	0.815	−0.76	−0.76	−0.765	−0.894
NPQ	0.032	0.24	−0.296	0.21	0.21	0.217	0.44
qN	−0.747	−0.591	0.544	−0.616	−0.616	−0.61	−0.408
qP	0.622	0.445	−0.392	0.473	0.473	0.466	0.246
qL	0.563	0.378	−0.324	0.407	0.407	0.4	0.174
ETR	0.879	0.761	−0.722	0.781	0.781	0.776	0.607

RAP: rapidly available potassium; AN: alkaline nitrogen; OM: organic matter; OC: organic carbon; TN: total nitrogen; CN: C/N ratio. Fv/Fm: maximum photosynthetic quantum yield of PS II, reflecting the photosynthetic potential of the sample. Fo: minimum fluorescence yield. Fm: maximum fluorescence yield. F: actual fluorescence intensity at any time. Fo’: minimum fluorescence yield under light. Fm’: maximum fluorescence yield under light. ^~^Fo’: minimum fluorescence under light. Y(II): actual photosynthetic quantum yield of PS II. Y(NPQ): the quantum yield of regulatory energy dissipation. Y(NO): the quantum yield of nonregulatory energy dissipation. NPQ: non-photochemical quenching coefficient. qN: non-photochemical quenching coefficient. qP: photochemical quenching coefficient. qL: photochemical quenching coefficient. ETR: electron transfer rate through PS II. * or ** indicate statistically significant differences; * *p* < 0.05; ** *p* < 0.01.

## Data Availability

The datasets used and/or analyzed during the current study are available from the corresponding author upon reasonable request.
